# Resection of Talocalcaneal Coalition With Calcaneal Lengthening Osteotomy: Short-to-Intermediate-Term Results

**DOI:** 10.7759/cureus.72846

**Published:** 2024-11-01

**Authors:** Michael Zaidman, Eden Weisstub, Vladimir Goldman, Naum Simanovsky

**Affiliations:** 1 Department of Orthopedic Surgery, Hadassah Hebrew University Medical Center, Jerusalem, ISR

**Keywords:** calcaneal lengthening osteotomy, coalition resection, functional outcome, pediatric population, radiological outcome, tarsal coalition

## Abstract

Introduction: The optimal surgical strategy for symptomatic tarsal coalition in pediatric patients remains debated. This study assessed the clinical and radiographic outcomes of addressing symptomatic talocalcaneal coalition through a combination of coalition resection and calcaneal lengthening osteotomy.

Materials and methods: We retrospectively reviewed cases of 10 children (11 feet) with symptomatic talocalcaneal tarsal coalition and painful flat feet who were treated between 2017 and 2019. All underwent calcaneal lengthening osteotomy (CLO) and coalition resection (RC). In half of the cases, the Achilles tendon was lengthened, and two children underwent medial plication. We analyzed demographic, clinical, and radiographic data, including CT scans for coalition confirmation and joint evaluations. Pre- and postoperative radiographic measurements and American Orthopedic Foot and Ankle Society (AOFAS) Ankle-Hindfoot Score assessed outcomes.

Results: The cohort, averaging 13.9 years at surgery, showed significant deformity correction. At follow-up (mean 54 months), six children were pain-free, and four experienced mild pain after intense activities. AOFAS scores were excellent or good. Complications included one superficial infection and mild forefoot supination in two children. No additional surgeries were needed.

Conclusion: Resection of talocalcaneal coalition combined with CLO effectively corrects rigid flat foot and alleviates pain, providing reliable outcomes in symptomatic cases.

## Introduction

Historically, the pathologic connection between two or more tarsal bones has been known as calcaneal bar, talocalcaneal bar, peroneal spastic flat foot, or rigid flat foot; today, the term tarsal coalition is widely accepted [1-3]. The talocalcaneal coalition is one of the two sites most affected. The coalition typically involves the whole middle facet, and in advanced cases, the coalition may consist of a complete bone bridge. Although non-surgical treatment is well-described [4], the optimal algorithm of operative management of symptomatic tarsal coalition is still under discussion in pediatric literature, and it is debated which surgical steps are essential to achieve the best outcome [5,6]. Isolated resection of coalition was generally preferred in the past. When resection is performed, a gap rather than a real joint is produced. In the best case, some degree of freedom of motion at the newly formed fibrous connection is achieved, but in the worst case, the new fibrous tissue may be rigid, or a solid bone bridge may reform. A more recent approach recommends not only resection of coalition but also the correction of the often co-occurring flat foot deformity by calcaneal osteotomy [6,7]. It is not yet clear whether resection of coalition alone or calcaneal osteotomy is more important. One may intuitively decide in favor of a combination of these two procedures to increase the likelihood of the best possible outcome. Today we have a better understanding of foot biomechanics [8-10], but the decision-making process about the best surgical approach can nevertheless be difficult and the algorithm for the best treatment is still evolving [6]. In this study, we aimed to assess the clinical and radiographic outcomes of the treatment of symptomatic talocalcaneal coalition by combined coalition resection (RC) and calcaneal lengthening osteotomy (CLO).

## Materials and methods

This study is approved by the Institutional Review Board of Hadassah Hebrew University. We retrospectively reviewed medical records, radiographs, and computerized tomography (CT) scans of 12 children operatively treated in our institution by one or more of the authors for painful flat feet and symptomatic talocalcaneal coalition (TCC) between the years 2017 and 2019. Two patients were excluded from the study due to lack of sufficient follow-up. Ten patients (11 feet), seven boys and three girls with a mean age of 13.9 (range: 11-17) years, were included in the study (Table 1). Eight of the 10 patients were skeletally immature. None of the patients had prior operations on their feet. Prior to surgical treatment, all patients had undergone conservative treatment, including the use of soft shoe insoles with medial arch support and rocker-bottom footwear as first-line treatment, and/or the application of a short-leg walking cast for five to six weeks.

**Table 1:** Demographic, radiographic, and clinical data. R: right; L: left; M: male; F: female; ST: sinus tarsi; Abs: absent; ROM: range of motion; norm: normal

Case no. and side	Gender and age at surgery (years+ months)	Pain location	Coalition type	Status of posterior facet	Status of Chopart joint	Follow-up length (months)	Subtalar ROM pre/postop
1. R	M14+4	Diffuse	Fibrous	Norm	Narrow	44	Abs/limited
2. L	M13+10	ST+medial	Fibrous	Norm	Narrow	70	Abs/limited
3. L	F11+1	Medial	Fibrous	Norm	Normal	56	Abs/limited
3. R	F12+5	Medial	Fibrous	Norm	Normal	44	Abs/limited
4. L	M15+6	Diffuse	Bony	Narrow	Narrow	66	Abs/Abs
5. L	M17+2	ST	Fibrous	Norm	Narrow	50	Abs/limited
6. L	M14+6	Medial	Bony	Narrow	Normal	50	Abs/limited
7. L	M15+10	ST+medial	Bony	Norm	Normal	51	Abs/limited
8. R	F12+7	Medial	Bony	Narrow	Normal	60	Abs/limited
9. L	M14+9	ST+medial	Bony	Norm	Normal	60	Abs/limited
10. R	F11+7	Medial	Fibrous	Norm	Normal	45	Limited/limited

The main indication for surgical intervention was a painful stiff or rigid flat foot without consistent improvement during at least six months of conservative treatment. In this condition, the pain is usually localized over the medial foot around the coalition, sometimes with an additional area of pain over the sinus tarsi or distally to the lateral malleolus. Only one patient reported isolated sinus tarsi pain and subtalar motion was practically absent in all but one patient.

**Figure 1:**
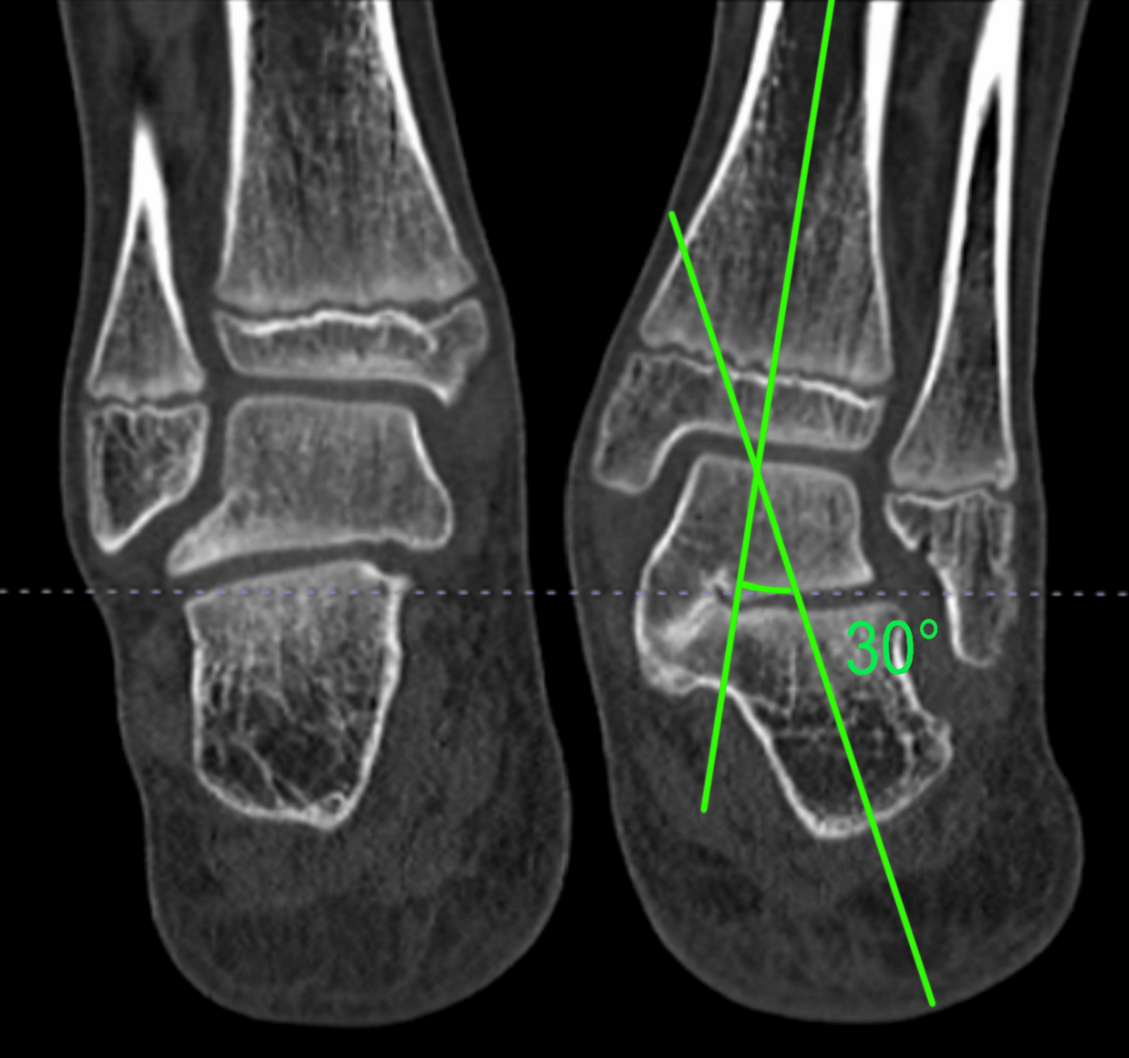
Measurement of hindfoot valgus on coronal reformats of CT. The left foot was normal, while the right foot exhibited flatfoot and a talocalcaneal coalition (TCC).

CT scans were used for radiographical confirmation of coalition and to evaluate the status of the posterior facet of the subtalar joint and transverse middle foot (Chopart) joints. Some authors recommend RC for those affecting less than 50% of the area of the posterior facet [11]. Other authors found no association between the size of TCC with respect to outcome scores, reporting favorable results when resections were performed on TCCs that were greater than 50% of the posterior facet [12,13]. In the present study, RC was carried out without calculation of coalition extension. Resection of coalition aimed to restore subtalar mobility and enable deformity correction by CLO. Measurements of hindfoot valgus were performed on coronal reconstructions of CT; more than 16° is considered of utmost value for isolated RC (Figure 1) [5,6,11,14]. The mean valgus hindfoot measured on coronal reformats was 29° (range: 21°-36°) (Table 2). The middle facet was completely obliterated on all feet. Five feet had a bony coalition, while six had a fibrocartilaginous coalition (Table 1). On CT scans, narrowing of the posterior facet was found in three feet and narrowing of the Chopart joint in four (Table 1). One patient had a narrowing of both joints. None of the patients met the criteria for isolated RC [13,15]. In 11 feet, combined RC and CLO were carried out. One patient was operated on both feet with an interval of 16 months.

**Table 2:** Preoperative and postoperative radiographic measurements and AOFAS Ankle-Hindfoot Score. CLO: calcaneal lengthening osteotomy; RC: resection of coalition; TAL: tendon Achilles lengthening; AOFAS: American Orthopedic Foot and Ankle Society Ankle-Hindfoot Score

Case no. and side	Talonavicular coverage angle pre/postoperative (degrees)	Calcaneal pitch angle pre/postoperative (degrees)	Meary’s angle pre/postoperative (degrees)	Hindfoot valgus preoperative (degrees)	Surgical procedure	Pain at last follow-up	Deformity correction	AOFAS score pre/postoperative
1. R	17/4	5/20	-3/2	33	CLO, RC, TAL	No	Complete	59/97
2. L	20/2	15/21	-2/2	31	CLO, RC, TAL	Rare, ankle	Complete	72/92
3. R	23/2	10/18	-5/2	24	CLO, RC, TAL	No	Complete	55/93
3. L	32/0	7/20	-6/0	21	CLO, RC, TAL	Rare, foot	Complete	59/90
4. L	15/1	13/22	0/4	35	CLO, RC, TAL	Rare, foot	Complete	58/87
5. L	23/2	6/15	-7/4	27	CLO, RC	No	Mild flat	69/94
6. L	10/2	13/23	0/6	33	CLO, RC	Rare, foot	Complete	62/92
7. L	30/1	15/22	-10/5	36	CLO, RC	No	Complete	64/97
8. R	27/3	11/18	-2/6	25	CLO, RC	No	Mild flat	59/94
9. L	20/0	10/20	-4/0	27	CLO, RC	No	Mild flat	58/95
10. R	25/5	5/20	-10/7	30	CLO, RC, TAL	No	Complete	62/94
Average (p-value)	22/2 (p<0.0001)	10/19.9 (p<0.0001)	-4.45/3.45 (p<0.0002)	-	-	-	-	61.55/93.18 (p<0.0001)

**Figure 2:**
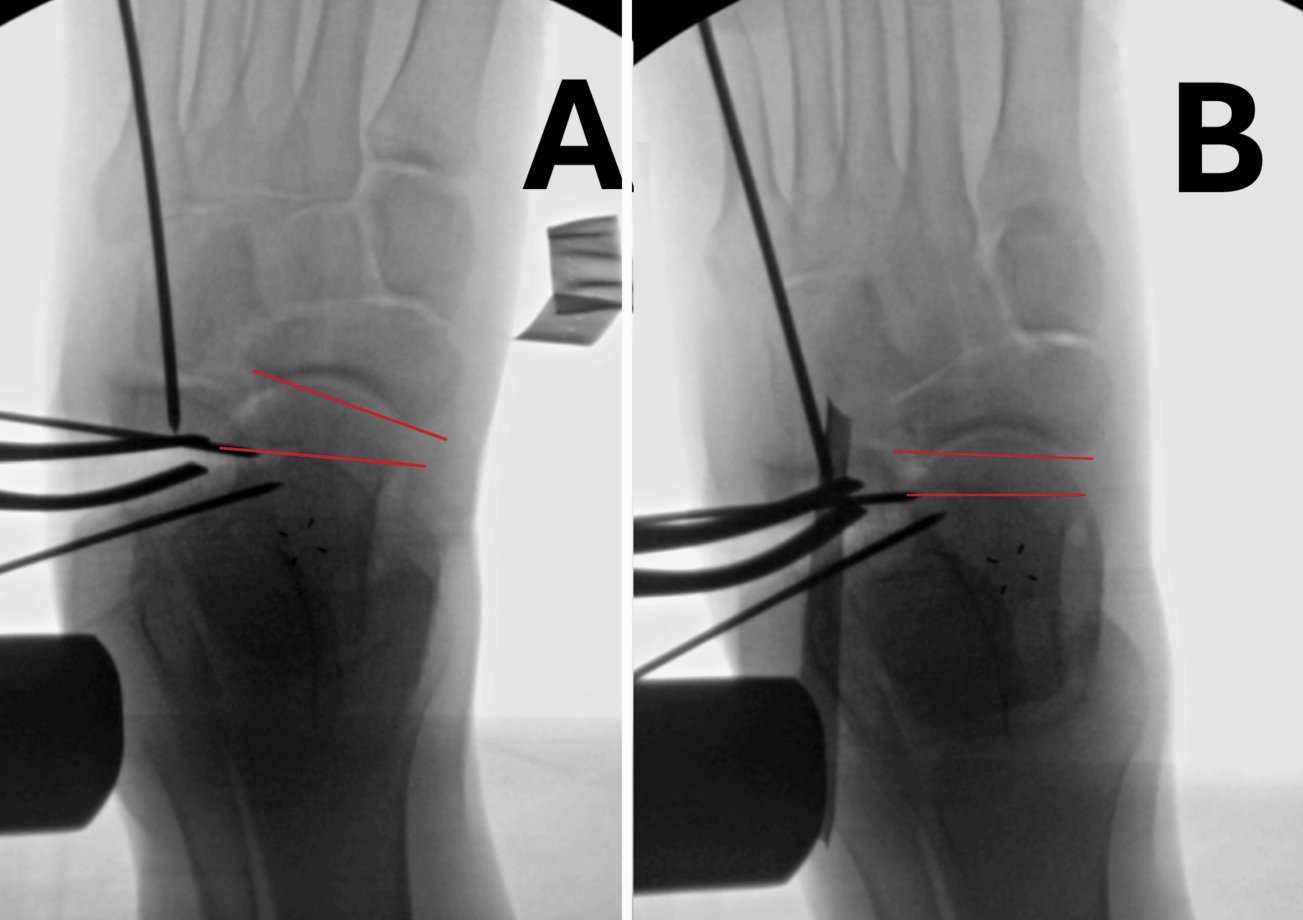
Intraoperative x-ray images showing correction of talonavicular coverage angle. (A) During osteotomy distraction before applying dorsiflexion and eversion stress. (B) The joint is under stress, with red lines indicating the articular surfaces of the talar head and navicular.

The authors preferred the operative technique. The surgeries were performed using the well-known CLO surgical technique described by Mosca, with the use of a tourniquet and C-arm assistance [15,16]. Medial plication was performed in two children; we found that, after deformity correction, the medial arch in most operated feet has sufficient stability - stress applied intraoperatively in the direction of dorsiflexion and eversion on an already-corrected foot did not produce longitudinal arc collapse. Adequate stability under stress was also confirmed by x-ray with the laminar spreader still inside the open calcaneal osteotomy, as the talonavicular angle remained unchanged (Figures 2A, 2B). Capsulotomy of the dorsolateral talonavicular joint was performed in two feet, only when obvious tightness was observed during distraction of calcaneal osteotomy. We routinely performed z-lengthening of the peroneus brevis tendon and release of the aponeurosis of the abductor digiti minimi.

Our usual source of bone graft for calcaneal osteotomy was the ipsilateral iliac crest. When it was technically possible, we used the meta-diaphyseal block from the ipsilateral distal fibula as a bone graft. Twice we used tricortical allografts. The options for graft sources were presented to and discussed with the families, and the decisions were made considering the families’ preferences.

To fill the gap remaining after the resection of the coalition, we used structured fat from the upper thigh, harvested from the grafting site, or a substantial fat pad from the sinus tarsi. Bone wax was usually added over the bone surfaces of the resected coalition. Our preferred way of fixation of calcaneocuboid joint and calcaneal osteotomy was transcuboid by one 2 mm smooth Kirschner wire. In six feet, gastrocnemius recession was added at the end of the surgery. The decision to proceed with gastrocnemius recession was based on ankle dorsiflexion at the end of the procedure. In case the ankle dorsiflexion exceeded the neutral position, no lengthening was performed. Due to concerns regarding possible inadvertent overlengthening and weakening, only half of the patients underwent gastrocnemius recession. We implemented Strayer or Vulpius techniques, depending on surgeon preferences. No cuneiform bone osteotomies were performed; however, mild residual asymptomatic flexible forefoot supination was observed in two patients at the last follow-up.

After surgery, a short-leg U-slab cast was applied for one week and then changed in the outpatient clinic for a short-leg fiberglass cast for another five to seven weeks. Toe touch weight bearing was permitted from the second week, and partial weight bearing was allowed from the fifth week until cast removal. The Kirschner wire was usually removed after five to six weeks in outpatient clinic.

**Figure 3:**
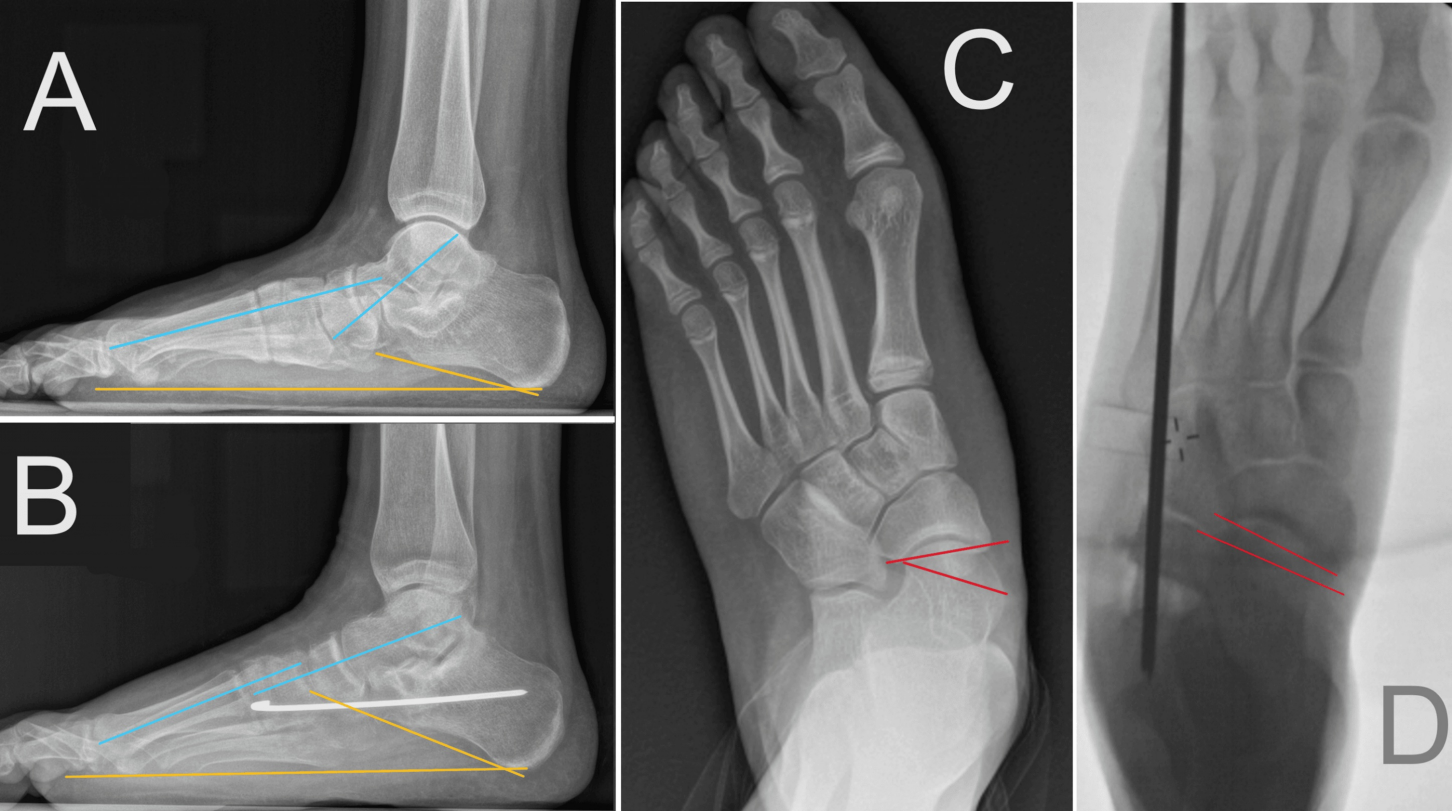
Foot alignment in pre-, intra-, and postoperative imaging. (A, B) Meary's angle (light blue lines) and pitch angle (orange lines), standing (A) preoperative and (B) postoperative views. (C, D) Talonavicular coverage angle measurements (red lines follow articular surfaces), (C) preoperative and (D) intraoperative views.

Demographic, clinical, and radiographic data were collected. Preoperative and postoperative anteroposterior and lateral weight-bearing radiographic measurements were compared (Figures 3A-3D and Table 2). To quantify degrees of foot deformity, we used calcaneal pitch angle (normal range: 18°-32°), lateral talar-first metatarsal (Meary's) angle (normal range: -4° to +4°), and talonavicular coverage angle (normal <7°) [17-20]. The clinical results were assessed using the American Orthopedic Foot and Ankle Society (AOFAS) Ankle-Hindfoot Score. The score is rated as excellent (90-100 points), good (80-90 points), fair (70-80 points), or poor (<70 points) [21]. We compare preoperatively estimated scores with postoperative results. Paired Student’s t-test was used for statistical analysis.

## Results

The mean follow-up was 54 (range: 44-70) months (Table 1). Only three patients presented with mild postoperative hindfoot valgus with some residual flat foot at the last follow-up. All radiographic parameters were normalized or considerably improved (Table 2). The preoperative mean talonavicular coverage angle was 22° (range: 32°-10°) as compared with the postoperative mean angle of 2° (range: 0°-5°) (p<0.0001). The calcaneal pitch angle improved from a mean of 10° (range: 5°-15°) to 20° (range: 15°-23°) (p<0.0001). The mean preoperative Meary’s angle of -4.5° (range: -10°-0°) improved to mean 3.5° (range: 0°-7°) (p<0.0002).

All feet displayed considerable functional improvement. In 10 of 11 feet, overall clinical outcome judged by AOFAS was excellent with a mean of 94 (range: 90-97) points, compared to preoperative mean score 62 (range: 58-72) (p<0.0001) (Table 2). One foot had an estimated postoperative score of 87 (Table 2). This patient initially presented with some narrowing of both the posterior facet and the Chopart joint and had a solid bony coalition. This patient was the only one who did not improve his subtalar range of motion, whereas all other 10 feet demonstrated limited but improved subtalar motion (Table 1). At the last follow-up, only four patients complained of some discomfort or mild intermittent pain over the operated area that mostly appeared after long-distance walking or running; they reported no functional limitations on regular activities. Early complications included one case of superficial wound infection and dehiscence, both resolved with oral antibiotic therapy. Late complications included two children with residual mild forefoot supination. None of the patients required additional operations during follow-up.

## Discussion

It is widely accepted to begin treatment of symptomatic TCC with non-operative means, such as shoe modifications, shoe insoles, physiotherapy, and activity adjustments. Unfortunately, in our experience, these attempts often fail to achieve consistent improvement, because steady obliteration of the middle facet of the subtalar joint progressively matures and solidifies. The initial limitation of the subtalar range of motion steadily progresses, and the foot becomes stiff or rigid. As a child grows and matures, the stress over the deformed foot rises accordingly, and adaptive changes around the subtalar and Chopart joints may result in a painful spastic flat foot [20]. The complex biomechanics of the normal and deformed foot were recently described in a comprehensive review by Ghanem et al. [10].

In the surgical treatment of painful flatfoot and TCC, determining the optimal approach is complex. Our experience suggests that isolated RC does not consistently provide pain relief. Furthermore, previously published studies indicate that isolated RC does not adequately correct stiff or rigid flatfoot [21-23]. Mosca and Bevan emphasize that addressing the underlying foot deformity is just as important as coalition resection in relieving pain associated with rigid flatfeet [6]. Yildiz et al. reported that all patients developed subtalar joint osteoarthritis within five years following simple tarsal coalition resection [24]. Similarly, Lisella et al. and Mahan et al. both concluded that residual foot deformity after isolated coalition resection can result in persistent pain and functional impairment. In contrast, hindfoot reconstruction combined with coalition resection has been shown to significantly improve joint motion, correct malalignment, and provide substantial pain relief [7,25]. The authors suggest that coalition resection and concomitant hindfoot reconstruction is a better option than resection alone. Gougoulias et al. observed that patients with preexisting hindfoot deformities who undergo isolated tarsal coalition resection may experience recurrent pain and worsening foot planovalgus [26]. Similarly, Lemley et al. suggest that increased hindfoot valgus may predict poorer outcomes when coalition resection is performed without addressing the underlying foot deformity [14]. Catanzano et al. emphasize that key outcomes, such as pain relief and the potential future need for arthrodesis, are influenced not only by the resection of the coalition but also by the correction of the deformity [20]. Additionally, Hetsroni et al. found that normal foot kinematics are not restored following tarsal coalition resection [27]. Following resection of a tarsal coalition, patients continue to be subjected to increased loading in their subtalar and adjacent articulations. This may result in further articular deterioration in the long term. He advises that additional operative procedures should be done to improve foot kinematics. Hetsroni et al. evaluated plantar pressure distribution after bar resection and found that normal plantar pressures are not restored following the resection of the tarsal coalition during recreational activity. This may have implications for abnormal foot loading and torque, potentially promoting degenerative changes in the subtalar and adjacent joints [28]. Skwara et al. reported fair outcomes, with a mean AOFAS score of 78.1, following simple RC [29]. Gait analysis revealed alterations in kinematic and kinetic parameters for the operated foot. Pedobarographic analysis showed altered loadings for the heel and forefoot. In his study, operative treatment of tarsal coalition achieved fair clinical and radiographic results and did not restore physiologic gait and foot loading.

X-ray and CT findings often do not meet established criteria for isolated RC [11,30]. For example, in all patients in this study, the hindfoot valgus was above acceptable limits for isolated RC, requiring the consideration of additional procedures. Stiff and valgus foot with malalignment of the Chopart joint is probably an important source of pain and mechanical abnormalities of the calcaneopedal unit (CPU). The biomechanics of the CPU are well-described [8]. In TCC, these biomechanics are disrupted due to the rigid connection between the talus and calcaneus. In the case of a fibrous coalition, RC without correction of the associated stiff flat foot could be counterproductive as it may lead to the formation of a bony coalition and the persistence of rigid symptomatic flat foot. The literature is sparse regarding the comparison of functional and radiographic outcomes of coalition resection alone versus coalition resection combined with foot deformity correction. Personally, we do not feel ethically and clinically correct to carry on coalition resection leaving significant deformities untreated.

Isolated CLO has been demonstrated as successful in correcting idiopathic flexible flat feet. Dumontier et al. demonstrated that almost all correction in CLO happens distally to the osteotomy site [9]. The questions are as follows: how can a corrective effect be achieved in the presence of TCC if all the changes take place distally to the site of the calcaneal osteotomy? What happens to the calcaneal body during the distraction process? Mosca explained that correction of the posterior CPU happens indirectly when the anterior part of the CPU is corrected directly during CLO [15]. However, isolated CLO is generally inadequate in the presence of a coalition, because the calcaneal body is rigidly connected to the talus during distraction of the osteotomy and lengthening of the calcaneus. The valgus position of the calcaneus considerably decreases during the opening of the osteotomy, with significant lengthening of calcaneus, the calcaneal body and tuberosity become corrected and stay in the neutral position. As the rigidly connected calcaneus and talus move together, this neutral position of the calcaneal body may lead to changes in the position of the talar dome, which may be forcefully pushed into a varus position. As a result, the tibial plafond and talar dome may become incongruent, and shearing stress may take place inside the talo-tibiofibular unit [10]. Therefore, the mechanics described by Dumontier et al. and Mosca will hold for a CLO only after resection of the coalition to enable correction of the calcaneus valgus position independently of the talus [9,15]. Therefore, in the presence of a coalition and stiff or rigid flat foot, the coalition must be resected prior to CLO. In this work, we present some of our intraoperative observations on the biomechanics of distraction of osteotomy for flat feet associated with TCC. First, during the distraction of osteotomy, the calcaneal body rotates externally in the horizontal plane around the vertical axis, causing the calcaneal tuberosity to shift from a lateralized and more horizontal position to a more neutral and vertical position. Additionally, the anterior part of the talus rotates externally as a conjoined calcaneo talar unit, and the talar head shifts to a more lateral position, further improving the talonavicular coverage angle. Second, the tension in the plantar fascia and the short toe flexors decreases talonavicular sag, so Meary's angle is normalized. The calcaneal body rotates in the sagittal plane around the horizontal axis, again together with the talus, increasing calcaneal pitch. At this point, any present triceps surae shortening can be uncovered, making obvious a previously masked equinus foot position. The addition of RC to CLO reveals a third intraoperative corrective effect, which occurs posterior to the calcaneal osteotomy - the disconnection of the fibrous or bony bridge between the calcaneus and the talus allows the calcaneal body to rotate independently from the talus around the talus in the coronal plane. This independent rotation enables true tilting of the calcaneus from a valgus into a more neutral position, which can be observed intraoperatively in the closure of the gap that appears after RC and in the convergence of the previously parallel-placed Kirschner wires in the talus and calcaneus. In other words, the addition of RC allows unobstructed varus movement of the calcaneal body during the distraction of calcaneal osteotomy. We opine that this calcaneal tilting also alleviates varus stress from the ankle joint that may persist if the coalition is left unresected, which is why we considered combining RC with CLO and presume that the effectiveness of CLO can be increased by the addition of RC. In the present study that combination results in deformity correction with favorable clinical outcome.

However, RC can probably be spared in cases when the TCC is still flexible enough to permit some subtalar motion, which may happen in less symptomatic younger patients. In these cases, CLO alone may be effective and prevent the development of rigid flat feet in the future. We suggest introducing more aggressive imaging evaluation, such as CT and MRI, of less-symptomatic feet in younger children in daily practice, to allow for an earlier diagnosis of TCC when CLO alone may be sufficient and effective.

There are several limitations to the study. First, it is a retrospective case series describing our results of combined coalition resection and deformity correction via CLO. Another limitation is its small sample size. Additionally, long-term follow-up is required.

## Conclusions

For symptomatic stiff or rigid flat feet associated with talocalcaneal coalition, the combination of RC and CLO consistently corrects all elements of rigid or stiff flat foot and reliably relieves pain. In our opinion, correction of deformities takes place distally and proximally to the calcaneal osteotomy; three corrective effects proximal to the calcaneal osteotomy were described. The combination of RC and CLO enables the achievement of unobstructed corrective tilt of the calcaneus during the surgery and improves the biomechanics of the CPU and talo-tibiofibular units. Therefore, the combination of CLO and RC is likely to be more effective than the isolated procedures for the treatment of TCC with stiff or rigid flat feet.
